# A Comparison of Dynamic Contrast-Enhanced Magnetic Resonance Imaging and T2-Weighted Imaging in Determining the Depth of Myometrial Invasion in Endometrial Carcinoma—A Retrospective Study

**DOI:** 10.3390/jpm12081268

**Published:** 2022-07-31

**Authors:** Idris Nurdillah, Iqbal Hussain Rizuana, Aziz Suraya, Sharis Osman Syazarina

**Affiliations:** Department of Radiology, Faculty of Medicine, Universiti Kebangsaan Malaysia Medical Center, Jalan Yaakob Latif, 56000 Kuala Lumpur, Malaysia; nu.della.65@gmail.com (I.N.); suraya.aziz@gmail.com (A.S.); syazasharis@yahoo.com (S.O.S.)

**Keywords:** DCE-MRI, T2WI, myometrial invasion, depth of myometrial invasion, endometrial carcinoma

## Abstract

This study aims to compare dynamic contrast-enhanced magnetic resonance imaging (DCE-MRI) with T2-weighted imaging (T2WI) in defining the depth of myometrial invasion in endometrial carcinoma. This retrospective study included 32 subjects with endometrial carcinoma who underwent 3.0T magnetic resonance imaging (MRI) prior to hysterectomy. DCE-MRI and T2WI were evaluated to determine the depth of myometrial invasion in endometrial carcinoma. A set of data consisting of the sensitivity, specificity, predictive values, and accuracy of DCE-MRI and T2WI were obtained and compared with the histopathological results. Out of the 32 cases included, the histopathological examination revealed that 50% myometrial invasion was found in 11 patients and ≥50% myometrial invasion was found in 21 patients. In the assessment of the tumor invasion, the sensitivity, specificity, positive predictive value (PPV), negative predictive value (NPV), and accuracy of T2WI were 45.45%, 90.48 %, 71.43%, 76.0%, and 75.0%, respectively. The corresponding values for DCE-MRI were 81.82%, 76.19%, 64.29%, 88.89 %, and 78.12%, respectively. When T2WI were read together with DCE-MRI, the values were 90.91%, 90.48%, 83.33%, 95.0%, and 90.62%, respectively. Thus, the sensitivity and accuracy of DCE-MRI were greater compared to T2WI in defining the depth of myometrial invasion. However, the merging of T2WI and DCE-MRI increased the specificity and PPV value and improved the sensitivity, NPV and accuracy in detecting myometrial invasion. DCE-MRI was more sensitive but less specific than T2WI in defining the depth of myometrial invasion. In conclusion, combining DCE-MRI and T2WI further improves the diagnostic performance for myometrial invasion in endometrial carcinoma.

## 1. Introduction

Endometrial carcinoma is the commonest gynecological cancer in the United States and the fourth most common malignancy among females worldwide [1]. In 2007, endometrial carcinoma was the sixth commonest cancer among women in Peninsular Malaysia, with 414 new cases registered under the Malaysian Cancer Statistics [2].

Endometrial carcinoma is conventionally surgically staged, and the stage of endometrial carcinoma is closely related to treatment planning and patient survival and prognosis. It is known that transvaginal ultrasonography (TVS) has a better performance trend in detecting cervical tumor spread. Therefore, TVS is ideal as the first-line imaging modality in patients with endometrial carcinoma [3]. Magnetic resonance imaging (MRI) plays a critical role in the preoperative planning, disease staging and therapy planning. Preoperative information about the depth of myometrial invasion and histopathological grade is essential in customizing the surgical approach for these patients. The evolution of MRI has been remarkably helpful in diagnosing gynecological cancers during the past two decades. To date, there is substantial evidence that MRI is useful for evaluating malignant conditions of the pelvis and known to be an excellent modality for evaluating the local stage of endometrial carcinoma, especially the depth of myometrial invasion [3]. Various studies reported that the overall staging accuracy of MRI were between 85% and 95% [4,5,6,7,8]. MRI has been found to be the only modality that accurately assesses myometrial, cervical, and nodal involvements in patients with a high risk of disseminated disease [3]. Due to its distinctive features of non-invasiveness, high soft tissue resolution, and multiple imaging parameters, it provides anatomical and functional information about the endometrial carcinoma and is playing an increasing role in clinical decisions regarding endometrial carcinoma [9]. 

The commonly used MRI protocols for assessing gynecologic tumors include T1-weighted images of the pelvis in the axial plane and T2-weighted images in the axial and sagittal planes. Fat-suppressed T1-weighted images facilitate the differentiation between fat and hemorrhage, which can both have a high signal intensity on T1-weighted images. For the staging of endometrial malignancies, T2-weighted imaging (T2WI), which can be used to assess the depth of myometrial invasion, and diffusion-weighted MRI (DWI), which differentiates between benign and malignant or metastatic lesions, are performed. In addition, T2WI with or without contrast-enhanced MRI (CE-MRI) can be used to stage endometrial carcinoma [10]. 

DCE-MRI quantifies the pharmacokinetic profile of an injected contrast agent with consecutive rapid image acquisition before, during, and after administration. The passage of the paramagnetic agent increases the signal intensity. The degree of enhancement is determined by the blood flow, vascular density, capillary permeability, and capillary surface area in the early vascular phase, and by extravascular space volume in the interstitial phase. Using DCE-MRI, endometrial lesions were detected at different phases, and the accuracy of the staging of endometrial carcinomas was increased compared to using T2WI (86.4% vs. 81.8%) [11,12]. 

## 2. Materials and Methods

### 2.1. Ethical Considerations

This retrospective study was approved by the institutional review board. Informed consent was waived, as this was a retrospective study (ethical approval code: FF-2017-506).

### 2.2. Patient Selection

Over a period of 36 months, 33 subjects consecutively underwent a total abdominal hysterectomy and bilateral salpingo-oophorectomy (TAHBSO) for the treatment of endometrial carcinoma, and subsequent histopathological diagnoses were obtained for the tumors at our hospital. This study only included patients who underwent TAHBSO. Patients who had undergone chemotherapy, radiotherapy, or hormonal treatment were excluded from this study. 

All the subjects had a pelvic MRI performed prior to hysterectomy. One subject was excluded due to missing pathological data. The remaining 32 patients (mean age, 59.8 years; range, 42–79 years), including 23 postmenopausal women (mean age, 63.5 years; range, 50–79 years) and 9 premenopausal women (mean age, 50.9 years; range, 42–56 years), were included in this study.

### 2.3. MRI Protocol

All MRI studies were performed using a 3.0T magnet (Siemens Magnetom Verio 3T MRI System, Siemens AG, Munich, Germany) equipped with a 16-channel HD body array coil. Patients fasted for at least 3 hours prior to the examination, and antiperistalsis agent with 20 mg hyoscine butyl bromide (Buscopan, Duopharma, Kuala Lumpur, Malaysia) was administered intravenously (IV) before the image acquisition to reduce bowel motion. 

Patients were instructed to empty their bladders before scanning in order to minimize motion due to bladder peristalsis and patient discomfort. The disease-specific protocol for patients with endometrial cancer (following the long axis of the uterus) included the following sequences (Table 1): (a) sagittal and coronal T2-weighted BLADE imaging, (b) axial oblique turbo spin-echo T2-weighted imaging of the uterus (perpendicular to the endometrial cavity), (c) axial turbo spin-echo T1-weighted imaging, (d) axial diffusion-weighted imaging, (e) axial turbo spin-echo T1-weighted fat-suppressed imaging, (f) sagittal gadolinium-enhanced DCE-MRI with spoiled gradient-echo (Fast Low Angle Shot (FLASH)), and (g) axial and sagittal post-contrast turbo spin-echo T1-weighted fat-suppressed imaging. DCE-MRI scans were acquired after an intravenous bolus injection of 10mls gadobenate-dimeglumine (Multihance, Bracco, Milan, Italy) using an automatic power injector at a 2 mL/s flow rate followed by a flush with 30mL of 0.9% sterile saline solution.

The dynamic contrast-enhanced (DCE) images were obtained using the 3D FS gradient-echo (GRE) T1WI sequence, following the intravenous contrast administration of 0.1 mmol/kg gadolinium at the rate of 2–3 mL/s. The dynamic images were acquired in the sagittal plane. Scanning was performed pre-contrast injection and at 30, 60, 90, 120, and 150 s after the injection. This was followed by further axial plane and sagittal plane delays accounting for approximately 4 min after injection.

### 2.4. Image Analysis

The MRI images were interpreted independently by two senior radiologists (with more than 5 years of experience in pelvic MRI), who were not aware of the histopathological (HPE) findings. The T2WI and DCE-MRI images were divided into two separate files, which were randomly assigned to each radiologist to evaluate the depth of myometrial invasion as either less than half (<50%) of the myometrium or more than half (≥50%) of the myometrium, using the T2WI, DCE-MRI, and the combination of the T2WI and DCE-MRI data. Tumors confined to the endometrium with an intact junctional zone (JZ), intact subendometrial enhancement (SEE), or a smooth tumor-to-myometrium interface, as well as those invading the inner half of the myometrium (focal disruption of the JZ, interrupted SEE, or irregular tumor-to-myometrium interface with less than <50% myometrial involvement), were classified as having <50% myometrium invasion. Tumors that extended beyond the outer half of the myometrium were classified as having ≥50% myometrial invasion. 

For the T2WI, DCE-MRI, and T2WI+DCE-MRI, if there was agreement between the two readers on the depth of myometrial invasion for the same set of images and for the same subject, the result was recorded as the final outcome. However, if there was any discrepancy between the two readers, both readers then reviewed the T2WI, DCE-MRI, and T2WI+DCE-MRI images together to achieve a final consensus regarding the tumor stage that could be agreed on by both readers, and this result was recorded as the final outcome. 

The final results were then compared with the HPE findings, which were taken as the gold standard reference.

### 2.5. Reference Standard

All patients underwent standard surgery in line with the current International Federation of Gynecology and Obstetrics surgical histopathological staging system. The corresponding surgical histopathology (HPE) findings (tumor size, the depth of myometrial invasion, myometrial thickness, and presence of concomitant diseases such as uterine fibroids and adenomyosis) were available in all cases and constituted the standard of reference in this study.

### 2.6. Statistical Analysis

The sensitivity, specificity, positive predictive value (PPV), negative predictive value (NPV), and diagnostic accuracy of DCE-MRI and T2WI alone, as well as of the combination of T2WI and DCE-MRI, were calculated. Kappa statistics were measured to assess the agreement regarding the depth of myometrial invasion on T2WI, DCE-MRI, and T2WI+DCE-MRI, with the depth of invasion based on the standard of reference, i.e., HPE. All analyses were conducted on 1 September 2018 using a web-based statistical tool, https://www.medcalc.org/ (MedCalc Software Ltd., Ostend, Belgium) and SPSS^®^ Version 22.0 (IBM^®^, Armonk, NY, USA).

## 3. Results

Out of the 32 endometrial carcinoma patients, 10 presented with one or two concomitant diseases; eight had uterine fibroids, and two had uterine fibroids and adenomyosis. Among the 32 patients, 23 patients were postmenopausal women, and nine patients were premenopausal women. The histopathological results indicated that twenty-six patients (81.3%; n = 26) had endometrioid adenocarcinoma, two (6.3%; n = 2) had adenosquamous carcinoma, two (6.3%; n = 2) had adenocarcinoma with mucinous differentiation, and two (6.3%; n = 2) had adenocarcinoma with serous differentiation. Eleven patients (34.4%; n = 11) were classified as having superficial invasion (<50% myometrial invasion) and twenty-one patients (65.6%; n = 21) were classified as having deep invasion (≥50% myometrial invasion) upon the histopathological examination.

**Figure 1:**
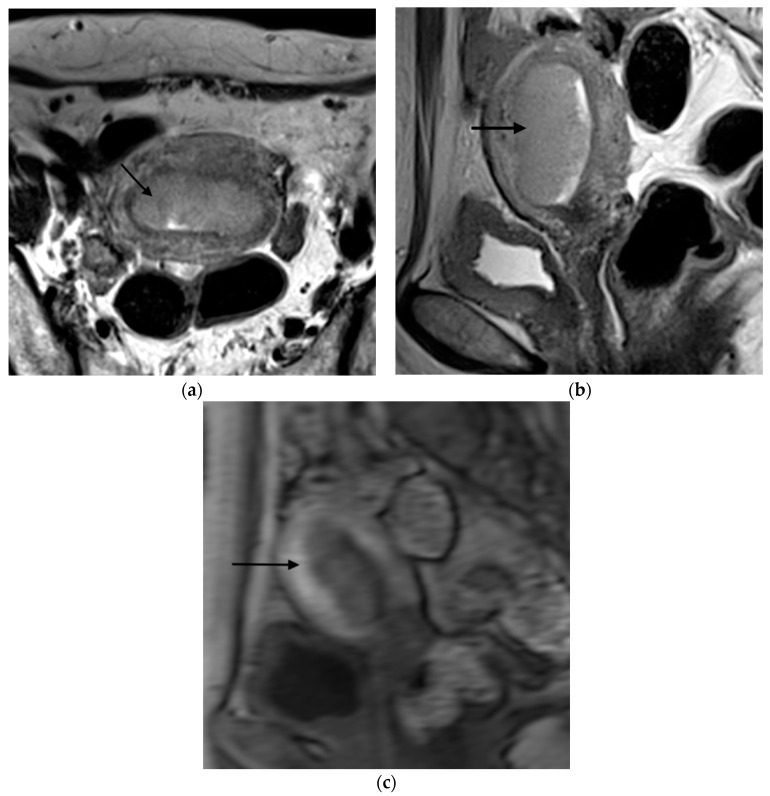
Representative MRI images of endometrial carcinoma with <50% myometrial invasion. (**a**) Axial T2WI, (**b**) sagittal T2WI, and (**c**) sagittal DCE-MRI images during the early acquisition stage. Tumors (arrow) with <50% myometrial invasion presented as iso- to mildly hyperintense on T2WI compared to the myometrium (**a**,**b**) with an intact junctional zone. On DCE-MRI, the tumors appeared as hypointense masses compared to the adjacent myometrium with intact subendometrial enhancement (SEE), indicating a tumor that is confined to the endometrium (**c**). Abbreviations: MRI, magnetic resonance imaging; dynamic contrast-enhanced magnetic resonance imaging (DCE-MRI); T2WI, T2-weighted imaging.

**Figure 2:**
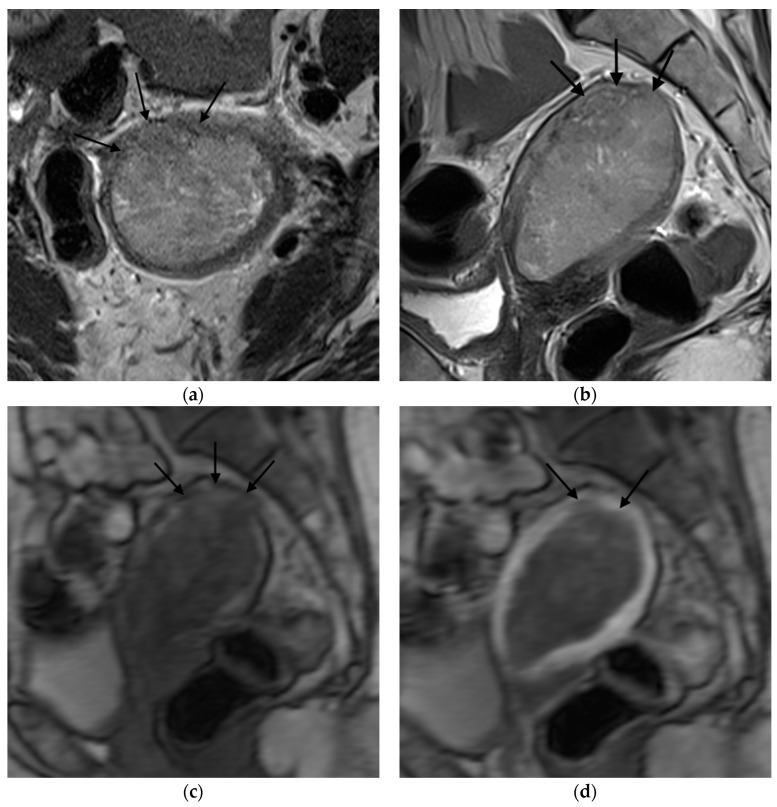
Representative MRI images of endometrial carcinoma with ≥50% myometrial invasion. (**a**) Axial T2WI, (**b**) sagittal T2WI, and (**c**,**d**) sagittal DCE-MRI images during the early (**c**) and late (**d**) acquisition stages. Tumors (arrow) with ≥50% myometrial invasion presented as iso- to mildly hyperintense on T2WI compared to the myometrium (**a**,**b**) with disruption of the junctional zone. On DCE-MRI, the tumors appeared as hypointense masses compared to the adjacent myometrium with interrupted subendometrial enhancement (SEE) (**c**), irregular tumor-to-myometrium interface, and extension of the tumor beyond the outer half of the myometrium (**d**). Abbreviations: MRI, magnetic resonance imaging; dynamic contrast-enhanced magnetic resonance imaging (DCE-MRI); T2WI, T2-weighted imaging; subendometrial enhancement (SEE).

**Figure 3:**
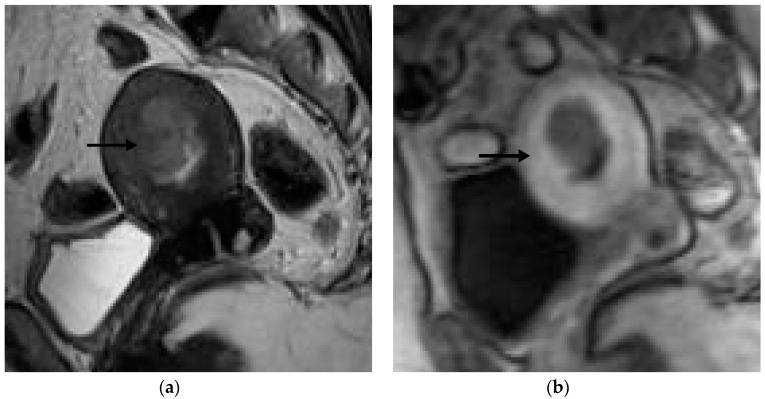
Representative MRI images of endometrial carcinoma of a false-negative case in a 50-year-old postmenopausal woman. (**a**) Sagittal T2WI MRI image shows a hyperintense lesion with blurring of the junctional zone (arrow). (**b**) DCE-MRI image shows a hypointense lesion with irregularity of the subendometrial enhancement (arrow) and <50% myometrial invasion. However, the histopathological result revealed ≥50% myometrial invasion.

**Figure 4:**
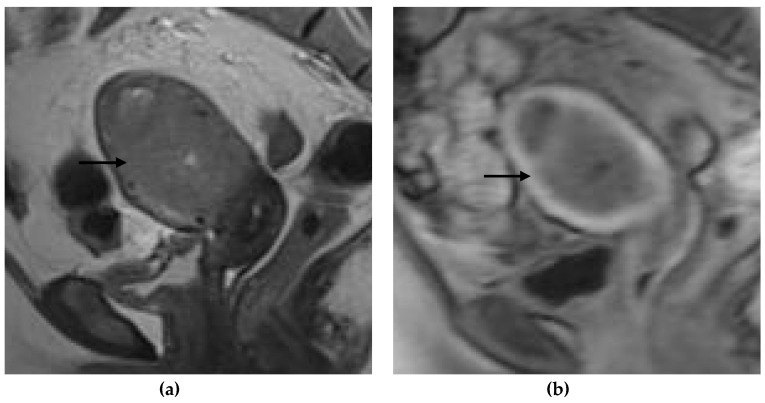
Representative MRI images of endometrial carcinoma of a false-positive case in a 67-year-old postmenopausal woman. (**a**) Sagittal T2WI MRI image shows a slightly hyperintense lesion with disruption of the junctional zone (arrow). (**b**) DCE-MRI image shows a hypointense lesion with thinning of the myometrium and ≥50% myometrial invasion. Subendometrial enhancement was not visible. However, the histopathological result revealed <50% myometrial invasion.

Figure 1, Figure 2, Figure 3 and Figure 4 depict the representative MRI images of tumors with < 50% myometrial invasion and ≥50% myometrial invasion, as well as the representative combined T2WI+DCE-MRI images for false-positive and false-negative cases, respectively. The diagnostic performance of MRI in predicting the depth of myometrial invasion is presented in Table 2 and Table 3. The depth of myometrial invasion (any depth) was correctly determined in 75% (n = 24) and 78% (n = 25) of cases on T2WI and DCE-MRI alone, respectively, whereas the percentage increased to 91% (n = 29) of cases when the T2W images were read together with those obtained by DCE-MRI.

On T2WI, five out of the 11 patients were correctly classified as having < 50% myometrial invasion and 19 out of 21 patients were correctly classified as having ≥50% myometrial invasion based on the pathological findings (Table 2). The sensitivity, specificity, PPV, NPV, and accuracy of T2WI in determining the depth of myometrial invasion were 45.45% (0.17–0.77), 90.48% (0.70–0.99), 71.43% (0.37–0.92), 76.00% (0.65–0.85), and 75.00%, respectively, with a kappa value of 0.39 (Table 3).

On DCE-MRI, nine out of the 11 patients were correctly classified as having < 50% myometrial invasion and 16 out of 21 patients were correctly classified as having ≥50% myometrial invasion based on the pathological findings (Table 2). The sensitivity, specificity, PPV, NPV, and accuracy of DCE in determining the depth of myometrial invasion were 81.82% (0.48–0.98), 76.19% (0.53–0.92), 64.29% (0.45–0.80), 88.89 % (0.69–0.97), and 78.12%, respectively, with a kappa value of 0.55 (Table 3).

When T2WI and DCE-MRI were read together, among the 11 patients with < 50% myometrial invasion of the tumor, 10 patients were correctly identified and 19 out of 21 patients were correctly classified as having ≥50% myometrial invasion (Table 2). The combination of T2WI and DCE-MRI produced sensitivity, specificity, PPV, NPV, and accuracy values of 90.91% (0.59–0.99), 90.48% (0.69–0.99), 83.33% (0.57–0.95), 95.00% (0.75–0.99), and 90.62% (0.75–0.98), respectively, with a kappa value of 0.80 (Table 3).

Table 4 shows the MRI performance in determining the depth of myometrial invasion in pre- and postmenopausal patients. The diagnostic accuracy in determining the depth of myometrial invasion in premenopausal patients on T2WI, DCE-MRI, and the combination of T2WI+DCE-MRI were 55.56%, 77.78%, and 88.89%, respectively. On the other hand, the diagnostic accuracy of T2WI, DCE-MRI, and the combination of T2WI+DCE-MRI in postmenopausal patients were 82.61%, 78.26%, and 91.30%, respectively (Table 4).

The sensitivity, NPV, and accuracy of DCE-MRI in determining the depth of myometrial invasion were superior to those of T2WI. However, when DCE images were read together with T2WI, this increased the specificity and PPV value and further improved the sensitivity, NPV, and accuracy in the detection of myometrial invasion.

## 4. Discussion

In this retrospective study, our results showed that DCE-MRI is more sensitive but less specific than T2WI. T2WI has a high specificity and low NPV in determining the depth of myometrial invasion, which indicates that T2WI alone can diagnose the absence of cancer growth in the myometrium among patients without pathological myometrial invasion. However, pathological myometrial invasion could not be excluded among the patients diagnosed by T2WI with the absence of myometrial invasion. In other words, it is difficult to completely eliminate microscopic myometrial invasion using MRI, especially based on T2WI alone. However, when T2WI and DCE-MRI were read together, the NPV values significantly improved, which indicates that the combination of T2WI and DCE-MRI can accurately determine the absence of myometrial invasion as compared to HPE. Our results also showed that combining DCE-MRI and T2WI resulted in a significant improvement in the diagnostic performance of MRI for the assessment of myometrial invasion. A prospective study by Manfredi et al. stressed the importance of multiphase dynamic MRI because it enabled the detection of different enhancement times of the endometrial tumor, distinguishing it from the adjacent normal myometrium [4]. The current study proved that using DCE-MRI (which can detect endometrial neoplasms at different phases) can increase the diagnostic accuracy in detecting the depth of myometrial invasion as compared to T2WI (78.12% vs. 75.0%), and this can be significantly improved with the combination of DCE-MRI and T2WI (90.62%). These results contradict Rockall et al.’s study on 84 patients, where the dynamic enhancement showed no improvement in the diagnostic performance [13]. However, this study was performed 10 years prior to the current study, during which MRI technology and protocols have improved significantly. Our findings concur with previous literature, where the routine use of DCE-MRI has significantly improved the accuracy of assessing the myometrial depth invasion (an accuracy of 55%-77% for T2W images vs. 85%-91% for DCE-MRI) [4,5,14,15,16]. We conclude that this combination of dynamic contrast-enhanced MRI with T2WI improved the NPV of myometrial invasion from 76.0% to 95.0% in our study.

The junctional zone (JZ) was found to be a notable landmark in defining the depth of myometrial invasion. On T2WIs, the correlation between the tumor and the JZ is highly predictive of deep myometrial infiltration [17]. Unfortunately, in patients with concomitant diseases (large endometrial tumors, adenomyosis, and multiple or large fibroids), a small or retroverted uterus, or a uterus with congenital anomalies, the zonal anatomical landmark may be difficult to identify, thus leading to the overstaging or understaging of the diagnosis [5,16,18]. DCE-MRI is a better tool compared to T2Wis in cases involving a thickened or ill-defined junctional zone, as it enables a clear differentiation between the tumor, endometrium, and myometrium. It is also more helpful in cases of large tumors causing a thinning of the myometrium. However, in a study performed by Rockall et al. involving a large series with 96 patients, there was no significant improvement in the performance of DCE-MRI over that of T2WI [13]. In the current study, out of 10 patients with concomitant diseases, five patients were incorrectly classified (either on T2WI, DCE-MRI, or a combination of T2WI and DCE-MRI). Amongst the five patients who were incorrectly classified, three patients were classified as having ≥50% myometrial invasion (overstaged) on T2WI, one was classified as having < 50% myometrial invasion (understaged) on DCE-MRI, and one was classified as having ≥50% myometrial invasion (overstaged) on both the T2WI and DCE-MRI sequences. However, none of the cases with concomitant diseases were incorrectly classified on the combination sequence of T2WI and DCE-MRI. Our results proved that combining T2WI and DCE-MRI can improve the detection of myometrial invasion in patients with concomitant diseases.

The presence and depth of myometrial infiltration can be assessed on T2WI as an interruption of the junctional zone, which appears as hypointense, contrary to endometrial adenocarcinoma, which appears as hyperintense. In postmenopausal patients, the junctional zone may be poorly visible, with the thinning of the myometrium due to uterine involution, making the presence and depth of myometrial infiltration more difficult to assess. In order to overcome this limitation, dynamic MRI is performed, because it can depict different enhancement times of the adenocarcinoma as distinguished from the adjacent myometrium, which improves the contrast resolution of the tumor and myometrium [4]. In our stratification analysis of premenopausal and postmenopausal subjects, we showed that the combination of T2WI and DCE-MRI offers better accuracy in defining the depth of myometrial invasion as compared to T2WI and DCE alone, in both pre- and postmenopausal subjects (88.89% and 91.3%). This is consistent with the study performed by Wu et al. [19]. Our results also showed that T2WI has a higher accuracy in postmenopausal subjects (82.61% for T2WI vs. 78.26% for DCE), and DCE-MRI has a higher accuracy in premenopausal subjects (77.78% for DCE vs. 55.56% for T2WI). This stands in contrast with a study by Lee et al., which reported that the assessment of myometrial invasion became more accurate when T2WI was used preferentially in the premenopausal group, while DCE-MRI was used preferentially in the postmenopausal group [20]. This inconsistent finding is probably due to the fact that there were more postmenopausal subjects in our study as compared to their study, which had an almost equal number of pre- and postmenopausal cases. 

The interobserver agreement between DCE-MRI and the histopathological staging was moderate (kappa 0.55), which was noticeably higher than that of T2WI (fair = 0.39) and corresponds with a previous study suggesting that DCE-MRI is a reproducible method for evaluating the depth of myometrial invasion and the endometrial carcinoma stage [21]. Moreover, with respect to the combination of T2WI and DCE-MRI, which is commonly employed in everyday clinical practice, our results indicate that the agreement was reliable (kappa 0.8) for detecting the depth of myometrial invasion of endometrial cancer. 

Currently, transvaginal ultrasound (TVS) and magnetic resonance imaging (MRI) [22] are the commonest techniques used for preoperatively assessing the depth of myometrial invasion, but MRI is more preferable and is the modality of choice, as it has a higher specificity and sensitivity in assessing the depth of myometrial invasion. Recent meta-analyses have shown that TVS has a 78%–85% sensitivity and 82%–84% specificity for detecting deep MI [22], whereas MRI offers a sensitivity ranging from 81% to 90% and specificity ranging from 82% to 89%, depending on the technique used [23,24].

Amongst the MRI protocols used in previous studies are unenhanced MRI, contrast-enhanced MRI (CE-MRI), and dynamic contrast-enhanced MRI (DCE-MRI), where the accuracy, sensitivity, and specificity for myometrial invasion range from 75% to 98%, 77% to 100%, and 72% to 100%, respectively [10,11,12,25]. DCE-MRI was first demonstrated to improve the staging accuracy of MRI for endometrial cancer in the early 1990s. A study by Sironi S. et al stated that a tumor can be distinguished from the blood products and debris within the endometrial cavity based on the MRI enhancement patterns [14]. In the case of postmenopausal women, morphological imaging proved to be of limited value. Hence, the combining of diffusion-weighted (DWI) and DCE-MRI imaging played a pivotal role in assessing the depth of myometrial invasion. The diagnostic accuracy of conventional MRI in this context ranges from 55% to 77% [4]. Manfredi R. et al. and Sala E. et al. also found that the combination of DCE-MRI and T2WI offers a “one-stop” examination for endometrial cancer staging, and it is recommended in the guidelines of the European Society for Urological Research for endometrial cancer staging. Furthermore, combining dynamic contrast-enhanced images with T2-weighted images has a diagnostic accuracy of up to 98% for assessing myometrial invasion [4,10]. Multiple studies have shown that DCE-MRI can augment conventional T2WI in defining the depth of myometrial invasion. However, the role of DCE-MRI alone in staging endometrial cancer remains controversial when compared to the pathological specimens. This is mainly because of the average underestimation of the measurement of myometrial thickness with the use of T2WI and DCE-MRI, as well as the overestimation of tumor invasion with DCE-MRI, the combined effects of these pitfalls resulting in a higher tumor stage (the overestimation of the tumor stage) [9,11,14,26]. Therefore, because the depth of myometrial invasion (i.e., the cancer stage) strongly correlates with metastasis and prognosis, determining the optimal MRI protocol is imperative for assessing the endometrial cancer stage. We hypothesize that DCE-MRI is better than T2WI in defining the depth of myometrial invasion in endometrial carcinoma. This study aimed to determine the effectiveness of DCE-MRI and T2WI sequences in defining the depth of myometrial invasion in endometrial carcinoma.

The goal of MRI is to pre-operatively identify patients who would benefit from abdominopelvic lymph node dissection and adjuvant therapy while avoiding over-treating early-stage patients with unnecessary lymphadenectomies [4]. The depth of myometrial invasion depicted by MRI also determines the need for adjuvant radiotherapy in stage-1B disease. Computed tomography (CT) scan is still widely used as standard practice in our center for evaluating distant metastasis. However, CT has limitations in differentiating soft tissue to determine the local staging of endometrial carcinoma compared to MRI. Thus, MRI is the modality of choice for the preoperative staging of endometrial carcinoma due to its ability to accurately depict the depth of myometrial invasion, in correlation with the tumor grade, lymph node metastases, and overall patient survival [9]. In our center, MRI is performed on patients with a histopathological diagnosis of endometrial carcinoma for the preoperative staging and to aid in the treatment planning.

The histological grade and the stage of endometrial carcinoma correlate strongly with the prevalence of lymph node metastasis and with patient prognosis [9]. They also determine the treatment, as most of the patients with early-stage disease require minimally invasive surgery. The treatment of choice for patients with low-risk stage-T1a endometrial cancer is a total hysterectomy and bilateral salpingo-oophorectomy [17,22]. Patients with greater than 50% myometrial invasion have a six- to seven-fold higher prevalence of pelvic and para-aortic lymph node metastasis than those with less than 50% myometrial invasion [27]. Therefore, patients with high risk factors, such as stage-IB tumors (≥50% myometrial invasion), require pelvic and para-aortic lymph node dissection followed by adjuvant chemotherapy with or without radiation therapy. However, lymph node resection may be omitted for tumors limited to the endometrium or to < 50% myometrial invasion, because less than 1% of these patients have lymphatic spread to the pelvic and/or para-aortic areas, for which a total hysterectomy is adequate [17,28]. For these reasons, pre-surgical knowledge of the depth of myometrial invasion is vital for treatment planning. It is necessary for stage-IA (< 50% myometrial invasion) endometrial carcinoma directly prior to surgery. 

Apart from DCE and DWI, there are new emerging techniques that aim to improve the MRI diagnosis of gynecological pathologies. Texture analysis (TA) is a technique that provides quantitative information about the image pixel intensity and distribution (the subtle heterogeneity not perceptible to the naked eye), and, in recent years, it has been increasingly used in radiological research as part of radiomics, machine-learning, and artificial intelligence techniques in computer-aided diagnosis. By defined parameters, TA can offer information about tissue characteristics, and its utility has been proven, especially in the diagnosis, disease-severity, treatment-response, prediction, and prognosis of several tumor sites [29,30].

The present study had some limitations, of which the following must be acknowledged. One limitation of our study was the sample size, making subgroup analysis difficult. Large-scale multicenter studies using similar methods are required to increase the impact of this study and to validate our results. Secondly, our study group included more postmenopausal women than premenopausal women. A study by Wu et al. has demonstrated that the accuracy of the preoperative evaluation of deep myometrial invasion using MRI was higher among premenopausal women compared to postmenopausal women (88.57% vs. 74.19%) [23]. This discrepancy can be attributed to the differences in myometrial thickness between the premenopausal and postmenopausal women. Furthermore, other studies have suggested that CE-MRI possesses better accuracy in assessing the depth of myometrial tumor invasion in postmenopausal patients with early endometrial carcinoma, but that T2WI is more suitable for the staging of endometrial carcinoma in premenopausal patients [15]. 

The third limitation is that, during our data collection, we did not have a pathologist to assist with the histology grade analysis, which is important for the disease prognosis in endometrial cancer. The subject characteristics of patients with <50% and ࣙ50% myometrial invasion, respectively, were also not statistically analyzed.

Other limitations of DCE include its lack of widespread availability and long image acquisition time. Finally, we did not perform staging using other functional MRI techniques, such as DWI and DTI. Recently, reports in the literature on the potential applications of DWI and DTI for determining myometrial invasion have staggeringly increased [24]. The combined usage of these techniques has proven to be more precise and valuable for further studies of the preoperative staging of endometrial carcinoma.

## 5. Conclusions

In summary, DCE-MRI is more sensitive but less specific than T2WI for the evaluation of the depth of myometrial invasion. However, the addition of DCE-MRI to T2WI can lead to a significant improvement in the diagnostic performance and accuracy of the assessment of the depth of myometrial invasion, especially in the context of certain pitfalls, such as concomitant disease, as well as in the assessment of postmenopausal patients/cases. Thus, it can serve as a useful and reliable tool for the preoperative assessment/staging of endometrial carcinoma in order to tailor the best surgical treatment.

## Figures and Tables

**Table 1:** Detailed sequences and protocols of MRI of the pelvis used during the study period.

Sequence	Axial T1	Sagittal T2 Blade	Coronal T2	Axial Oblique T2	Axial DWI	Axial T1FS	Sagittal DCE	Axial Oblique DCE	Axial T1FS CE	Coronal T1FS CE
TE (ms)	12	94	94	97	82	11	0.9	0.9	11	11
TR (ms)	522	4000	4000	5610	7800	700	2.18	2.18	700	700
Echo train length		26	26	14						
Flip angle (degrees)		90	90	90						
FOV	220	230	230	210	260	200	300	300	200	200
Slice thickness (mm)		3	3	3	3	3	3	2	3	3
Matrix size		0.7 × 0.7 × 3.0 mm	0.7 × 0.7 × 3.0 mm	0.7 × 0.5 × 3.0 mm	2.3 × 1.8 × 3.0 mm	1 × 0.8 × 3.0 mm	1 × 0.8 × 3.0 mm	1 × 0.8 × 3.0 mm		
b values					0, 500, 800, 1000					

**Table 2:** Comparison of T2WI, DCE, and T2+DCE with HPE in determining the depth of myometrial invasion.

Depth of Myometrial Invasion on MRI		Depth of Myometrial Invasion in HPE		Total
		<50%	≥50%	
		n = 11 (34.4)	n = 21 (65.6)	n = 32 (100)
T2WI	<50%	5 (15.6) (TP)	2 (6.2) (FP)	7 (21.9)
	≥50%	6 (18.8) (FN)	19 (59.4) (TN)	25 (78.1)
DCE	<50%	9 (28.1) (TP)	5 (15.6) (FP)	14 (43.7)
	≥50%	2 (6.3) (FN)	16 (50.0) (TN)	18 (56.3)
T2 + DCE	<50%	10 (31.3) (TP)	2 (6.3) (FP)	12 (37.5)
	≥50%	1 (3.1) (FN)	19 (59.4) (TN)	20 (62.5)

Data are presented as n (%).

**Table 3:** Performance of the MRI sequences in determining the depth of myometrial invasion.

	Sensitivity	Specificity	PPV	NPV	Accuracy	Kappa
	(95% CI)	(95% CI)	(95% CI)	(95% CI)		
T2WI	45.45%	90.48%	71.43%	76.00%	75.00%	0.39
	(0.17–0.77)	(0.70–0.99)	(0.37–0.92)	(0.65–0.85)	(0.57–0.89)	
DCE	81.82%	76.19%	64.29%	88.89%	78.12%	0.55
	(0.48–0.98)	(0.53–0.92)	(0.45–0.80)	(0.69–0.97)	(0.60–0.91)	
T2 + DCE	90.91%	90.48%	83.33%	95.00%	90.62%	0.80
	(0.59–0.99)	(0.69–0.99)	(0.57–0.95)	(0.75–0.99)	(0.75–0.98)	

Abbreviations: MRI, magnetic resonance imaging; CI, confidence interval; PPV, positive predictive value; NPV, negative predictive value; DCE, dynamic contrast-enhanced; T2WI, T2-weighted imaging.

**Table 4:** Performance of the MRI (magnetic resonance imaging) sequences in determining the depth of myometrial invasion in pre- and postmenopausal states.

	Sensitivity	Specificity	PPV	NPV	Accuracy
	(95% CI)	(95% CI)	(95% CI)	(95% CI)	
Premenopausal					
T2WI	25.0%	80.00%	50.00%	57.14	55.56%
	(0.01–0.81)	(0.28–0.99)	(0.08–0.92)	(0.4–0.73)	(0.21–0.86)
DCE	75.00%	80.00%	75.00%	80.00%	77.78%
	(0.2–0.99)	(0.28–0.99)	(0.32–0.95)	(0.41–0.96)	(0.39–0.97)
T2 + DCE	100.00%	80.00%	80.00%	100.0%	88.89%
	(0.4–10.0)	(0.28–0.99)	(0.41–0.96)	-	(0.52–0.99)
Postmenopausal					
T2WI	57.14%	93.75%	80.00%	83.33%	82.61%
	(0.18–0.9)	(0.7–0.99)	(0.35–0.97)	(0.68–0.92)	(0.61–0.95)
DCE	85.71%	75.00%	60.00%	92.31%	78.26%
	(0.42–0.99)	(0.48–0.93)	(0.34–0.79)	(0.66–0.99)	(0.56–0.93)
T2 + DCE	85.71%	93.75%	85.71%	93.75%	91.30%
	(0.42–0.99)	(0.69–0.99)	(0.47–0.98)	(0.71–0.99)	(0.72–0.99)

Abbreviations: MRI, magnetic resonance imaging; CI, confidence interval; PPV, positive predictive value; NPV, negative predictive value; DCE, dynamic contrast-enhanced; T2WI, T2-weighted imaging.

## Data Availability

The datasets generated during and/or analyzed during the current study are not publicly available due to patients’ privacy but are available from the authors on reasonable request.
